# Statins Prevent the Deleterious Consequences of Placental Chemerin Upregulation in Preeclampsia

**DOI:** 10.1161/HYPERTENSIONAHA.123.22457

**Published:** 2024-02-16

**Authors:** Lunbo Tan, Ans C.M. Kluivers, Edwyn O. Cruz-López, Michelle Broekhuizen, Zhongli Chen, Rugina I. Neuman, Sam Schoenmakers, Liesbeth Ruijgrok, Daan van de Velde, Brenda C.M. de Winter, Antoon J. van den Bogaerdt, Xifeng Lu, A.H. Jan Danser, Koen Verdonk

**Affiliations:** 1 Division of Vascular Medicine and Pharmacology, Department of Internal Medicine (L.T., A.C.M.K., E.O.C.-L., M.B., R.I.N., A.H.J.D., K.V.), Erasmus MC, Rotterdam, the Netherlands.; 2 Department of Obstetrics and Gynecology (A.C.M.K., R.I.N., S.S.), Erasmus MC, Rotterdam, the Netherlands.; 3 Division of Neonatology, Department of Neonatal and Pediatric Intensive Care (M.B.), Erasmus MC, Rotterdam, the Netherlands.; 4 Department of Internal Medicine, Academic Center for Thyroid Diseases (Z.C.), Erasmus MC, Rotterdam, the Netherlands.; 5 Department of Hospital Pharmacy (L.R., D.v.d.V., B.C.M.d.W.), Erasmus MC, Rotterdam, the Netherlands.; 6 Clinical Research Center, The First Affiliated Hospital of Shantou University Medical College, China (L.T., X.L.).; 7 Heart Valve Department, Euro Tissue Bank-Bio Implant Services LIFE (ETB-BISLIFE), Beverwijk, the Netherlands (A.J.v.d.B.).

**Keywords:** fluvastatin, placenta growth factor, pravastatin, preeclampsia, pregnancy

## Abstract

**BACKGROUND:**

Chemerin, an inflammatory adipokine, is upregulated in preeclampsia, and its placental overexpression results in preeclampsia-like symptoms in mice. Statins may lower chemerin.

**METHODS:**

Chemerin was determined in a prospective cohort study in women suspected of preeclampsia and evaluated as a predictor versus the sFlt-1 (soluble fms-like tyrosine kinase-1)/PlGF (placental growth factor) ratio. Chemerin release was studied in perfused placentas and placental explants with or without the statins pravastatin and fluvastatin. We also addressed statin placental passage and the effects of chemerin in chorionic plate arteries.

**RESULTS:**

Serum chemerin was elevated in women with preeclampsia, and its addition to a predictive model yielded significant effects on top of the sFlt-1/PlGF ratio to predict preeclampsia and its fetal complications. Perfused placentas and explants of preeclamptic women released more chemerin and sFlt-1 and less PlGF than those of healthy pregnant women. Statins reversed this. Both statins entered the fetal compartment, and the fetal/maternal concentration ratio of pravastatin was twice that of fluvastatin. Chemerin constricted plate arteries, and this was blocked by a chemerin receptor antagonist and pravastatin. Chemerin did not potentiate endothelin-1 in chorionic plate arteries. In explants, statins upregulated low-density lipoprotein receptor expression, which relies on the same transcription factor as chemerin, and NO release.

**CONCLUSIONS:**

Chemerin is a biomarker for preeclampsia, and statins both prevent its placental upregulation and effects, in an NO and low-density lipoprotein receptor–dependent manner. Combined with their capacity to improve the sFlt-1/PlGF ratio, this offers an attractive mechanism by which statins may prevent or treat preeclampsia.

**Novelty and Relevance**

**What Is New?**

In a predictive model, the addition of the adipokine chemerin improved the prediction of early onset preeclampsia and fetal complications to a similar degree as the sFlt-1 (soluble fms-like tyrosine kinase-1)/PlGF (placental growth factor) ratio, and when combined with this ratio, better prediction rates were achieved.
In a placental perfusion model, both pravastatin and fluvastatin transferred to the fetal compartment, and the fetal/maternal concentration ratio at steady state was twice as high for pravastatin.
Perfused placentas and explants of early onset preeclamptic women released more chemerin and sFlt-1 and less PlGF than those of healthy pregnant women, and statins reversed this. This effect relies most likely on the capacity of statins to upregulate NO and the low-density lipoprotein receptor.
Chemerin constricted chorionic plate arteries, and this was prevented by both pravastatin and the CCRL2 (chemerin chemokine-like receptor 2) antagonist α-NETA (2-[a-Naphthoyl]ethyltrimethylammonium iodide). Chemerin did not potentiate endothelin-1.

**What Is Relevant?**

Chemerin overexpression of the placenta yields preeclampsia-like symptoms in pregnant mice, while in women with preeclampsia, its levels are elevated and correlate with blood pressure and proteinuria. Ex vivo studies with human placentas, either perfused or being used as explants, confirm chemerin upregulation in early onset preeclampsia and additionally reveal that statins may downregulate chemerin on top of their capacity to lower sFlt-1. These drugs transfer to the fetal compartment and also block chemerin-induced vasoconstriction.

**Clinical/Pathophysiological Implications?**

Serum chemerin may help to identify women at high risk for preeclampsia and its fetal complications.
Statins transfer to the fetal compartment, and their transfer differs per statin.
By lowering both chemerin and sFlt-1, statins may offer a new treatment option for preeclampsia.

Preeclampsia is a multisystem hypertensive disorder that affects 5% to 8% of pregnancies, causing serious maternal and fetal morbidity and mortality.^1,2^ Preeclampsia is defined as a new onset of hypertension in the presence of either proteinuria, maternal organ dysfunction, or uteroplacental dysfunction.^1,2^ Unfortunately, its pathophysiological mechanism remains unclear, and it is neither predictable nor preventable during the early stages of pregnancy.^3^ As a result, preeclampsia management relies on symptom management, rather than treatment. It is, however, known that women with preeclampsia have an imbalance of angiogenic markers in their blood, characterized by an elevated sFlt-1 (soluble fms-like tyrosine kinase-1) concentration and a decreased PlGF (placental growth factor) concentration. The sFlt-1/PlGF ratio is a tool that is increasingly used to predict preeclampsia and its complications.^4,5^ When combined with maternal demographic characteristics and medical history, it can enhance the sensitivity of prediction in preeclampsia. However, there are still many cases of misdiagnosis.^4,5^

Chemerin is an adipokine that, under nonpregnant conditions, is primarily secreted by the liver and adipose tissue, contributing to multiple metabolic and inflammatory processes.^6–8^ Chemerin synthesis and secretion have been linked to the low-density lipoprotein receptor (LDLR), and the expression of both chemerin and the LDLR is regulated by the transcription factor SREBP2 (sterol regulatory element-binding protein 2).^9^ In pregnant women, circulating chemerin exhibits an increase in concentration during pregnancy and subsequently undergoes a rapid decline after delivery.^10–12^ Chemerin is involved in angiogenesis and spiral artery remodeling and might trigger delivery.^13,14^ However, it may also play a role in the development of preeclampsia, given that its concentrations in blood correlate positively with the severity of preeclampsia and adverse fetal outcomes.^14,15^ In agreement with this concept, placental overexpression of chemerin in pregnant mice induced a preeclampsia-like syndrome, involving high blood pressure, proteinuria, endothelial dysfunction, fetal growth restriction, and an increased sFlt-1/PlGF ratio.^10^ Moreover, preeclamptic human placentas released more chemerin compared with healthy ones.^10^ These data suggest that placental release may underlie the increase in circulating chemerin during preeclampsia.

Chemerin exerts its actions by binding to the CMKLR1 (chemerin chemokine-like receptor 1).^6^ A second receptor, CCRL2 (CC motif chemokine receptor-like 2), functions as a chaperone receptor, concentrating chemerin locally and thereby allowing optimal chemerin-CMKLR1 interaction.^6^ CMKLR1 activation is responsible for the deleterious effects observed in pregnant mice with placental chemerin overexpression.^10^ This receptor also upregulates constrictor responses.^16^ Consequently, it might be beneficial to reduce placental and circulating chemerin in preeclampsia.

Statins are lipid-lowering drugs that inhibit the activity of HMG-CoA (3-hydroxy-3-methylglutaryl coenzyme A) reductase. They simultaneously upregulate LDLR expression in the liver, thereby increasing the clearance of circulating LDL (low-density lipoprotein) cholesterol.^17^ Statins additionally induce anti-inflammatory effects, improve endothelial function,^17^ and lower circulating chemerin in patients with acute myocardial infarction.^18,19^ Statins have been proposed as therapeutic agents in the management of preeclampsia based on the benefits that they provide to patients with angiogenic imbalance, endothelial dysfunction, oxidative injury, and inflammatory injury.^20^ Evidence from animal studies, in vitro human studies, and small clinical trials (ranging from 12 to 30 weeks of gestation) has shown that statins, especially pravastatin, reduce the symptoms of preeclampsia, possibly by reducing placental sFlt-1 release.^17,21–23^ However, in a large trial, starting pravastatin treatment between 35 and 37 weeks of gestation did not reveal any effect of pravastatin on either circulating sFlt-1 or the incidence of preeclampsia.^3^ Here, a caveat is that many aspects of early onset preeclampsia might already have developed before 35 weeks. It is also not known to what degree statins are able to pass the placental barrier.

In the present study, we hypothesized first that chemerin might be a novel discriminating marker, either alone or on top of the sFlt-1/PlGF ratio, for preeclampsia and its related complications and second that statins might lower placental chemerin synthesis, thereby improving preeclampsia outcome. We, thus, assessed the value of chemerin versus the sFlt-1/PlGF ratio as a predictor, making use of an existing prospective multicenter cohort study in women with suspected or confirmed preeclampsia. In addition, by applying an ex vivo placenta perfusion model, we investigated the transplacental transfer of 2 statins, pravastatin, and fluvastatin, as well as their effects on the release of chemerin, sFlt-1, and PlGF from healthy and preeclamptic placentas. Finally, we studied the molecular mechanism underlying the effects of statins on chemerin release making use of placental villous explants, and we examined whether pravastatin blocks the vascular effects of chemerin in chorionic plate arteries.

## METHODS

### Data Availability

The data that support the findings of this study are available from the corresponding author upon reasonable request.

### Collection of Blood From Pregnant Women

We analyzed available residual serum samples from a previously conducted prospective cohort.^24^ All subjects provided written informed consent to participate in the study, which was approved by the local research ethics committee (MEC-2013-202).^24^ As described in the original study, women with singleton pregnancies with suspected or confirmed preeclampsia were recruited to analyze the use of the sFlt-1/PlGF ratio between 2013 and 2016 in 3 Dutch hospitals.^24^ Preeclampsia was defined as de novo hypertension (systolic blood pressure ≥140 mm Hg or diastolic blood pressure ≥90 mm Hg) and proteinuria (protein/creatinine ratio ≥30 mg/mmol or ≥300 mg/24 hour or 2+ dipstick) at or after 20 weeks of pregnancy, superimposed preeclampsia (chronic hypertension with new-onset proteinuria or preexisting proteinuria with new-onset hypertension) or hemolysis, elevated liver enzymes, and low platelet count syndrome according to the standard criteria.^24^ Women who were initially suspected of preeclampsia in whom the diagnosis was not confirmed were defined as having no preeclampsia. This group also included other hypertensive disorders of pregnancy. Only the samples of early onset preeclampsia (defined as the diagnosis of preeclampsia before 34 weeks of gestation) and no preeclampsia were included in the present study. This resulted in the use of 467 leftover serum samples of the original 620 women. Maternal complications were defined as eclampsia, development of (superimposed) preeclampsia or the hemolysis, elevated liver enzymes, and low platelet count syndrome after inclusion into the study, pulmonary edema, subcapsular liver hematoma, cerebral hemorrhage/edema or infarction, postpartum hemorrhage (blood loss ≥1000 mL after delivery), and acute renal failure (absolute increase in the serum creatinine concentration of ≥0.3 mg/dL [26.4 μmol/L] from baseline, ≥50% increase in serum creatinine, or oliguria with <0.5 mL/kg per hour for a period of 6 hours).^24^ All patients diagnosed with hemolysis, elevated liver enzymes, and low platelet count at initial inclusion were excluded from the calculation of maternal complications. Fetal complications were defined as fetal death, neonatal (birth) weight <10th percentile according to Perinatal Registration, the Netherlands, development of sepsis, admittance to the neonatal intensive care unit, artificial ventilation and its duration, bronchopulmonary dysplasia (defined as chronic lung disease developing in preterm neonates treated with oxygen and positive-pressure ventilation, with radiographic signs of inflammation and scarring, in need of artificial ventilation 4 weeks of postpartum and at 36 weeks of postmenstrual age), respiratory distress syndrome, necrotizing enterocolitis, intraventricular hemorrhage, periventricular leukomalacia, and posthemorrhagic ventricular dilatation.^24^ Days from enrollment to delivery were defined as days from blood sampling until delivery.

### Collection of Placentas From Pregnant Women

Placentas were collected from 41 women (23 healthy controls and 18 patients with early onset preeclampsia) with singleton pregnancies who underwent elective cesarean sections. This preeclampsia diagnosis was based on the International Society for the Study of Hypertension in Pregnancy 2018 criteria.^25^ Inclusion and exclusion criteria have been described previously.^26^ All women who donated their placenta gave approval through written informed consent, and the placentas were collected immediately after delivery at the Erasmus Medical Center, Rotterdam, the Netherlands, in the period from 2020 to 2023. Clinical characteristics were obtained from the electronic files of the patients. The medical ethical committee of the Erasmus MC waived the need for approval within the Dutch Law on research with humans (MEC-2016-418 and MEC-2017-418).

### Ex Vivo Dual-Sided Placental Cotyledon Perfusion

The perfusion model used in the current study has been described previously and is summarized in Figure S1A.^26,27^ In brief, both the maternal and fetal circulations of a placental cotyledon were restored and perfused with Krebs-Henseleit buffer (in mmol/L: NaCl, 118; KCl, 4.7; CaCl_2_, 2.5; MgSO_4_, 1.2; KH_2_PO_4_, 1.2; NaHCO_3_, 25; and glucose, 8.3) at 37 °C, supplemented with heparin (final concentration: 5000 IU/L), and oxygenated with a mixture of 95% O_2_ and 5% CO_2_. Maternal and fetal flow rates were set at 12 and 6 mL/min, respectively, and the perfusion was maintained as an open circulation for ≈45 minutes to remove blood from the cotyledon. Hereafter, 2 closed circulations were formed by letting the maternal and fetal outflows recirculate into their respective reservoirs. Both circulating buffers were then replaced with 200 mL fresh buffer, marking the starting point of the experiment (t=0). At t=0, the maternal circulation contained 1 mg/L of pravastatin (Sigma-Aldrich, Darmstadt, Germany), 5 mg/L of fluvastatin (Sigma-Aldrich), or no drug as a control. The used drug concentrations correspond with their peak blood levels in patients.^28,29^ Samples were collected from both maternal and fetal circulations at t=0, 6, and 30 minutes and subsequently every 30 minutes until the conclusion of the 180-minute perfusion experiment. Samples were stored at −80 °C until further analysis. For the positive transfer control, antipyrine (100 mg/L, Sigma-Aldrich) was added to the maternal circulation at t=0. Simultaneously, fluorescein isothiocyanate-dextran (40 kDa, 36 mg/L, Sigma-Aldrich) was added into the fetal circulation to assess the integrity of the capillary bed. To evaluate the success of the experiment, the fetal-to-maternal ratio of antipyrine had to exceed 0.75 at t=180, and the maternal-to-fetal ratio of fluorescein isothiocyanate-dextran had to remain below 0.03 throughout the experiment.

### Liquid Chromatography-Mass Spectrometry Analysis of Pravastatin and Fluvastatin

A validated ultraperformance liquid chromatography–tandem mass spectrometry method was used to measure fluvastatin and pravastatin concentrations in plasma. In this simultaneous quantification method, atorvastatin-d5 (Cayman Chemical, Ann Arbor, MI) and pravastatin-d3 (Cayman Chemical) were used as internal standards for fluvastatin (Cayman Chemical) and pravastatin (Cayman Chemical). Quantification was operated in multiple reaction monitoring modes, and electrospray ionization was set in the positive mode for fluvastatin and the negative mode for pravastatin. Chromatographic separation was performed on a C18 column (50 °C) using a gradient of methanol water containing 0.1% formic acid and 0.013% ammonium acetate and a flow rate of 0.4 mL/min. The range of detection was 5.5 to 451 µg/L for fluvastatin and 6 to 430 µg/L for pravastatin, with correlation coefficients of 0.997 and 0.998, respectively. The precision was tested at 3 different concentration levels in the detection range and has a relative SD of 4.3% to 11.3% for fluvastatin and 5.0% to 10.2% for pravastatin. The method is validated according to the US Food and Drug Administration guidance on bioanalytical method validation. All maternal samples were diluted 10× to measure in the concentration range. The transfer ratio of each sampling time point (fetal/maternal ratio) was calculated by dividing the concentration of pravastatin or fluvastatin in the fetal circulation by that in the maternal circulation.

### Placental Villous Explant Culture

The placental villous explant culture protocol used in the current study has been described before and is summarized in Figure S1B.^30^ In short, immediately after collection of the placenta, 3 full-thickness slices were dissected from the tissues, the chorionic and basal plates were removed, and the remaining central villous tissue was transferred to a sterile tube and stored on ice. The central villous tissue was thoroughly washed with ice-cold PBS and cut into 2 × 2 mm explants. Three explants from the 3 different slices were combined into a 12-well plate with 2 mL Dulbecco's Modified Eagle Medium/F12 medium (Lonza, Breda, the Netherlands) supplemented with 10% fetal bovine serum (GE Healthcare, Eindhoven, the Netherlands), 1.95 g/L NaHCO_3_, and 100-mg/L Primocin (InvivoGen, San Diego, CA). The placental villous explants were left to equilibrate for 3 hours at 37 °C, 8% O_2_, and 5% CO_2_. Thereafter, the villous explants were transferred into wells with fresh medium and exposed to either of the following treatments: (1) no drug as control; (2) 20 and 200 μmol/L fluvastatin; or (3) 20, 200, and 2000 μmol/L pravastatin.^23,31^ After 24 hours, the wet weight of explants was recorded, the culture medium was collected, and the tissues and medium were stored at −80 °C until further analysis.

#### Western Blotting

Placental villous explants were homogenized in RIPA buffer (150 mmol/L NaCl, 1% triton X-100, 0.5% sodium deoxycholate, 0.1% sodium dodecyl-sulfate, 50 mmol/L Tris, and pH 8.0) with protease and phosphatase inhibitors. After incubating on ice for 10 minutes, the samples were centrifuged at 4 °C and 12 000 g for 10 minutes. The supernatant was collected from each sample, and the total protein concentration was quantified using the BCA assay kit (Thermo Fisher Scientific, Waltham, MA). For immunoblotting analysis, explant lysates containing an equal amount of protein (15 μg) were loaded onto a 4% to 20% midi protein gel (Bio-Rad Laboratories, Hercules, CA) and resolved through sodium dodecyl-sulfate polyacrylamide gel electrophoresis. The proteins were then transferred to the membrane using a constant current of 2.5 A for up to 25 V for 7 minutes, using the Trans-Blot Turbo System (Bio-Rad Laboratories). Afterward, the membranes were blocked in Tris-buffered saline with 5% skimmed milk (Carl Roth, Karlsruhe, Germany) for 1 hour. The membranes were incubated with primary antibodies, as detailed in Table S1, overnight at 4 °C. After washing with Tris-buffered saline with 0.1% Tween-20, horseradish peroxidase-conjugated goat anti-mouse or goat anti-rabbit antibodies (Bio-Rad Laboratories) were added and incubated at room temperature for 1 hour. The membranes were then washed again for chemiluminescence analysis. Chemiluminescence analysis was detected with enhanced chemiluminescence (ECL; Bio-Rad Laboratories). The Western blot images were quantified using Image J. Protein abundances were normalized versus GAPDH (glyceraldehyde 3-phosphate dehydrogenase) and are presented as a percentage of the average normalized ratio of the control group.

Given the existence of multiple isoforms in the case of both chemerin and PlGF,^8,10,32–36^ we used human retinoic acid receptor responder 2 small interfering RNA (small interfering-Chemerin, 10 nmol/L; Thermo Fisher Scientific, 12179), and human PlGF siRNA (small interfering-PlGF, 50 nmol/L; Thermo Fisher Scientific, 143789) to knockdown chemerin and PlGF in HepG2 cells (ATCC, Manassas, VA) and placenta villous explants.^37,38^ A nontargeting siRNA (small interfering control) was used as a negative control (Thermo Fisher Scientific, AM4611). HepG2 cells were cultured in a complete medium with Eagle’s Minimum Essential Medium (ATCC) and 10% fetal bovine serum (GE Healthcare) in T-75 flasks (Costar, Corning, NY) at 37 °C with 5% CO_2_. The medium was refreshed every 2 to 3 days, and the cells were subcultured at 80% to 85% confluence. Explants were cultured as described above. siRNA was added to the culture medium with transfection reagents according to the instructions of the manufacturer (Thermo Fisher Scientific, 13778075). After 24 hours, explants and HepG2 cells were collected for Western blotting using the serum of preeclamptic and nonpreeclamptic patients as a positive control. Figure S2A shows that chemerin knockdown of chemerin in the explant concerned the bands below 25 kD and above 15 kD when using the Abcam antibody, while the Proteintech antibody yielded a band at 25 kD, only which was not affected by the knockdown. On this basis, in all further studies, we used the band above 15 kD as the representative band for chemerin. PlGF yielded a single band (between 55 and 70 kD) in the Hep2G cells and multiple bands in the explants (Figure S2B). Knockdown in both situations diminished all bands, and on this basis, we used the average of the bands between 55 and 70 kD as the representative band for PlGF.

#### mRNA Analysis

Total RNA from the placental villous explant lysates was extracted using a commercial kit (Zymo Research, Irvine, CA) according to the instructions of the manufacturer and reverse transcribed using PrimeScript RT Master Mix for real-time PCR (Takara Shuzo, Kyoto, Japan), at 37 °C for 15 minutes, followed by 85 °C for 5 s, and 4 °C for further analysis. Real-time PCR was performed using the PowerTrack SYBR Green Master Mix (Thermo Fisher) according to the instructions of the manufacturer. We used the following mix: 0.1 µL of yellow sample buffer, 5 µL of PowerTrack SYBR Green master mix, 0.6 µL of forward and reverse primer (10 ng/µL), 0.4 µL of nuclease-free Milli-Q water, and 4 µL of DNA template to a final volume of 10 µL. The qPCR was performed with the CFX Opus 384 Real-Time PCR System (Bio-Rad Laboratories) with the following steps: initial denaturation at 95 °C for 2 minutes followed by 40 cycles of denaturation at 95 °C for 15 s and annealing extension at 60 °C for 1 minute and final melt curve stage where the temperature ramped up from 60 °C to 95 °C increasing by 1 °C every 0.15 s. Primer sequences are listed in Table S2. Expression levels were determined using a standard curve, and human 36B4 (acidic ribosomal phosphoprotein P0), β-actin, and YWHAZ (tyrosine 3-monooxygenase) were used as an internal control to normalize gene expression levels.

### Wire Myography Experiments

Second-order branches of chorionic plate arteries of both healthy and preeclamptic placentas were identified, carefully dissected, cleaned from surrounding tissue, stored overnight in Krebs-Henseleit buffer at 4 °C, and oxygenated with a mixture of 95% O_2_ and 5% CO_2_. The vessels were cut into 2 mm segments and mounted in organ baths (Danish Myograph Technology, Aarhus, Denmark) containing 6 mL Krebs-Henseleit buffer at 37 °C and aerated with a mixture of 95% O_2_ and 5% CO_2_. In chorionic plate arteries, the tension was normalized to 90% of the estimated diameter at 38 mm Hg effective transmural pressure to mimic the physiological circumstances of placental vessels. When the segments reached a stable baseline pressure, the maximum generated force (here referred to as contractile response) to 100 mmol/L KCl was determined. Only segments with a KCl response >3 mN were included. After washout of KCl, concentration-response curves were constructed to either chemerin-9 (the active isoform of chemerin; MedChemExpress) or ET-1 (endothelin-1, Sigma-Aldrich) with or without preincubated with one of the following compounds for 30 minutes: 3 μmol/L α-NETA (2-[a-Naphthoyl] ethyltrimethylammonium iodide, MedChemExpress) or 20 μmol/L pravastatin (Sigma-Aldrich). In the case of ET-1, preincubations with 10 nmol/L chemerin-9 were also included.

### Biochemical Measurements

Chemerin concentration in serum samples (maximum 400× dilution in kit reagent), perfusate samples (30× dilution in the case of maternal perfusates and no dilution in the case of fetal perfusates), and explant culture medium (20× dilution) was assessed by a commercial ELISA according to the guidelines of the manufacturer (R&D Systems DY2324). The detection limit was 31.2 pg/mL. Measurements of sFlt-1 and free PlGF in the prospective cohort study had been performed earlier using the automated Elecsys immunoassay from Roche Diagnostics (Cobas e801 6000e; Roche Diagnostics, Almere, the Netherlands).^24^ The sFlt-1 concentration in perfusate samples and explant culture medium was assessed by a commercial ELISA according to the instructions of the manufacturer (R&D Systems DY321B). The detection limit was 125 pg/mL. PlGF in perfusate samples and explant culture medium was assessed by a commercial ELISA according to the instructions of the manufacturer (R&D Systems DY264). The detection limit was 31.2 pg/mL. Total PlGF was measured in the explant culture medium by placing the samples in a heating block at 70 °C for 10 minutes, as described previously.^39^ The hCG (human chorionic gonadotropin) concentrations in the explant culture medium were assessed by a commercial ELISA according to the instructions of the manufacturer (SinoBiological SEK10903). The detection limit was 5.47 pg/mL. The LDH (lactate dehydrogenase) concentrations in the explant culture medium were assessed by a commercial colorimetric kit according to the instructions of the manufacturer (Thermo Fisher C20300). The other serum results were obtained from digital medical files.

### Statistical Analysis

Data are presented as the mean (±SEM) for normally distributed continuous variables or median (interquartile range) for nonnormally distributed continuous variables and as a number (percentage) for categorical variables. To assess whether continuous variables exhibited a normal distribution, the Shapiro-Wilk normality test (n<50) or the Kolmogorov-Smirnov test (n≥50) test was utilized. To compare the 2 groups, the Student *t* test was used, or the Mann-Whitney *U* test was used in the case of nonnormally distributed data. When comparing continuous variables among more than 2 groups, a 1-way ANOVA was applied, or the Kruskal-Wallis test was applied in the case of nonparametric distributions, with the Dunnett correction for multiple testing being applied. Two-way ANOVA combined with the Sidak multiple comparison test was applied when evaluating the concentration-response curves of the wire myography experiments and the statin effects in explants. Spearman ρ was applied to calculate correlation coefficients between clinical parameters and serum chemerin. Pearson was applied to calculate correlation coefficients between chemerin concentrations in the explant tissue and medium. *P*<0.05 was considered statistically significant. The prediction models were compared using concordance (c)-statistic for the dichotomous outcomes. The statistical analysis was performed using GraphPad Prism (version 8.0, La Jolla, CA), R Software, version 4.0.5, and SPSS (version 27.0, SPSS, Chicago, IL) on Mac.

## RESULTS

### Clinical Characteristics

The clinical characteristics of 467 singleton pregnancies with suspected or confirmed preeclampsia are presented in Table S3. Of these women, 101 were confirmed to have early onset preeclampsia. No significant differences were found in age, sex, ethnicity, parity, prepregnancy body mass index (BMI), history of preeclampsia, and history of smoking between the patients with no preeclampsia and early onset preeclampsia. The gestational age at sampling and delivery, birthweight, and estimated glomerular filtration rate in the patients with confirmed preeclampsia were lower than those in the patients with no preeclampsia. The parity, sFlt-1/PlGF ratio, creatinine, uric acid, alanine aminotransferase, protein in the urine, mean arterial pressure, and chemerin concentrations in the patients with early onset preeclampsia were higher than those in the patients with no preeclampsia.

### Serum Chemerin Correlation With Clinical Parameters and Its Impact on the Prediction of Preeclampsia and Complications

The correlation between chemerin and clinical parameters is provided in Table S4. Chemerin concentrations positively correlated with the sFlt-1/PlGF ratio, creatinine, uric acid, proteinuria, and mean arterial pressure. Negative correlations were found with birthweight and estimated glomerular filtration rate. No significant correlation was observed with maternal age, prepregnancy BMI, and gestational age at sampling. Because the circulating sFlt-1/PlGF ratio and chemerin are correlated,^10^ we repeated our correlation analysis after correcting for the ratio. The outcome was the same, with the exception of the correlation with proteinuria, which was no longer significant.

**Figure 1.**
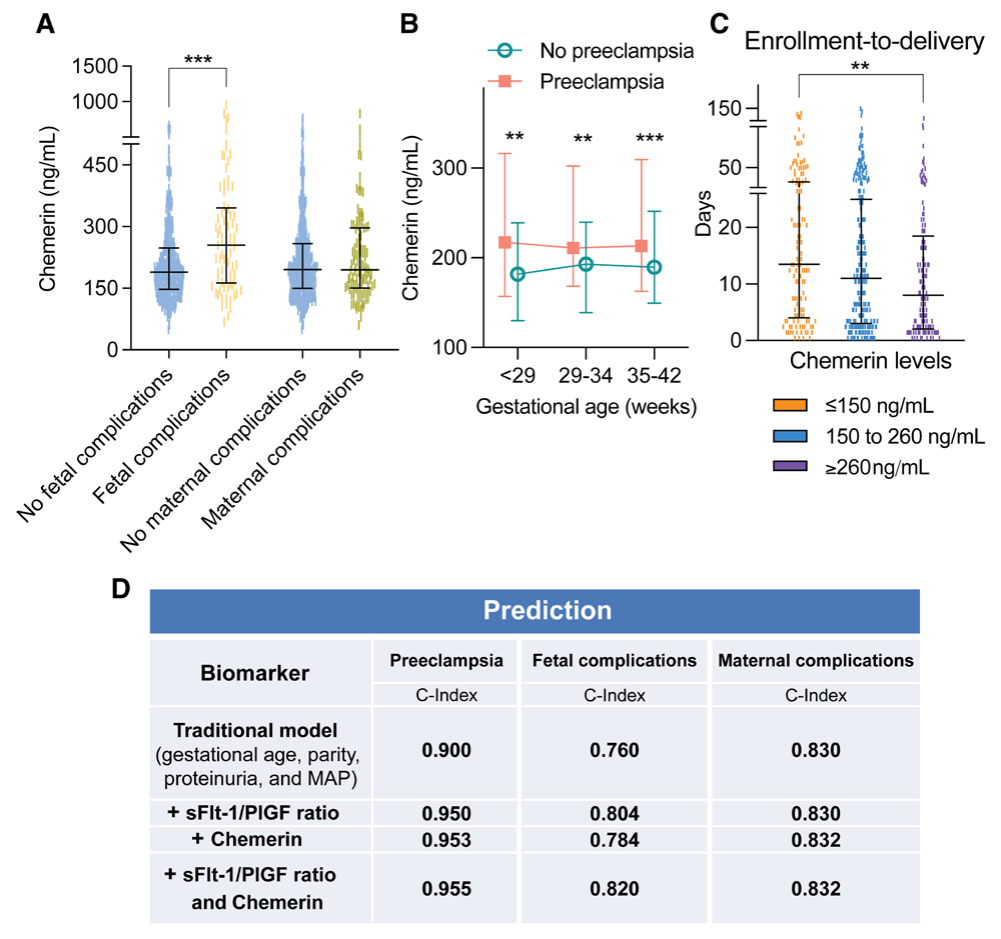
**Serum chemerin and its predictive value. A**, Serum chemerin levels according to the occurrence of fetal (361 without and 78 with) and maternal (322 without and 106 with) complications. Data are individual points plus median and interquartile range. ***P<0.001. **B**, Serum chemerin levels according to gestational age in women with (n=101) or without preeclampsia (n=366). Data are median and interquartile range. **P<0.01, ***P<0.001 vs no preeclampsia. **C**, Subdividing the entire population in tertiles according to serum chemerin levels reveals that high serum chemerin levels relate to a shorter enrollment-to-delivery time. Data are individual points plus median and interquartile range. **P<0.01. **D**, Predictive value of chemerin and the sFlt-1 (soluble fms-like tyrosine kinase-1)/placental growth factor (PlGF) ratio on top of the traditional model. MAP indicates mean arterial pressure.

Maternal chemerin was elevated in pregnancies with fetal complications but not in pregnancies with maternal complications (Figure 1A). Chemerin concentrations were higher in preeclamptic pregnancies at all gestational ages (Figure 1B). When dividing the cohort in tertiles according to serum chemerin levels (each having a median gestational age of 35 weeks), higher chemerin levels are associated with a shorter duration of pregnancy (Figure 1C).

Preeclampsia, fetal complications, and maternal complications can be predicted based on a model involving traditional parameters (gestational age, parity, proteinuria, and mean arterial blood pressure).^24^ Adding the sFlt-1/PlGF ratio to this traditional model improved the predictive value (C-index) for all outcomes, and the same was true for serum chemerin, while the highest predictive values were obtained when adding both the ratio and serum chemerin (Figure 1D).

### Placental Perfusion: Chemerin, sFlt-1, and PlGF Release During Pravastatin or Fluvastatin Exposure

We obtained 53 cotyledons from 47 pregnancies, of which during perfusion 31 met the quality control criteria, implying that the success rate was 59%, like in our previous studies.^27,40^ The clinical characteristics of these samples are given in Table S5.

**Figure 2.**
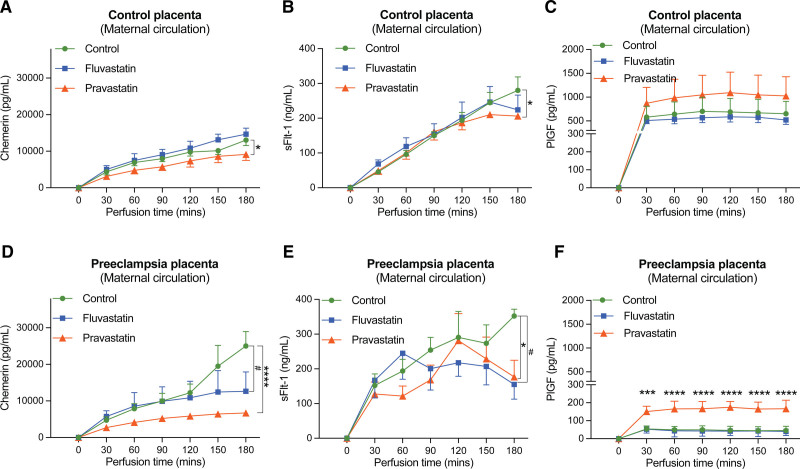
**Maternal release of chemerin, sFlt-1 (soluble fms-like tyrosine kinase-1), and PlGF (placental growth factor) from healthy and preeclamptic placentas during vehicle or statin perfusion. A** through **C**, Concentrations of chemerin, sFlt-1, and PlGF in the maternal effluent of healthy placentas perfused with vehicle (control; n=10), 5 mg/L fluvastatin (n=4), or 1 mg/L pravastatin (n=5). **D** through **F**, Concentrations of chemerin, sFlt-1, and PlGF in the maternal effluent of preeclamptic placentas perfused with vehicle (control; n=5), 5 mg/L fluvastatin (n=3), or 1 mg/L pravastatin (n=3). Data are mean±SEM. *P<0.05, ***P<0.001, and ****P<0.0001 pravastatin vs control at the same perfusion time. #*P*<0.05, fluvastatin vs control at the same perfusion time.

During the control perfusion of healthy placentas, chemerin and sFlt-1 levels increased over time in the maternal (Figure 2) and fetal perfusion fluid (Figure S3), while PlGF levels reached a plateau after 30 to 60 minutes in both compartments. In all cases, maternal levels were substantially (up to a 1000-fold) higher than fetal levels, indicating that the release of all 3 proteins occurred predominantly maternally. As expected, preeclamptic placentas released more chemerin and sFlt-1 and less PlGF (Figure S4A through S4C). These differences were only observed maternally.

**Figure 3.**
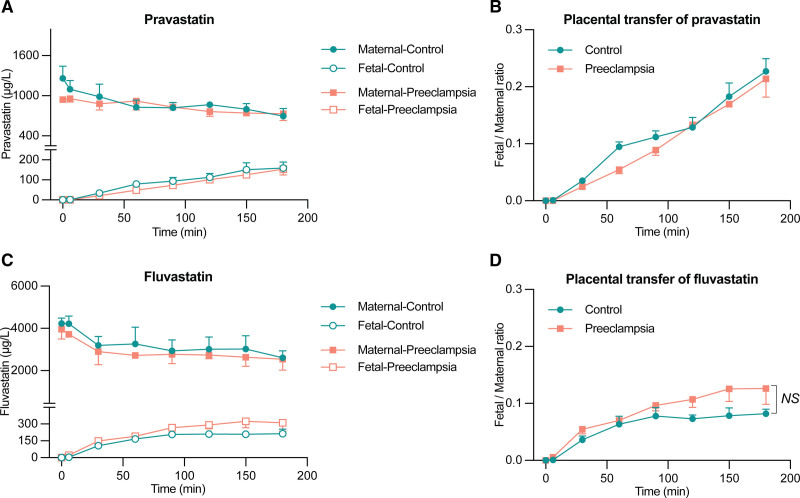
**Placental transfer of pravastatin and fluvastatin in healthy and preeclamptic pregnancies. A** and **B**, The concentration of pravastatin in the maternal and fetal circulation of healthy (n=5) and preeclamptic (n=3) placentas and the fetal/maternal concentration ratio of pravastatin. **C** and **D**, The concentration of fluvastatin in the maternal and fetal circulation of healthy (n=5) and preeclamptic (n=3) placentas and the fetal/maternal concentration ratio of fluvastatin. Data are mean±SEM. NS indicates not significant.

Figure 3 shows the transplacental transfer of pravastatin and fluvastatin. Neither drug affected the perfusion pressure. Both pravastatin and fluvastatin transferred from the maternal to the fetal circulation in healthy placentas, and the fetal/maternal concentration ratio at t=180 minutes for pravastatin was higher than that for fluvastatin (*P*<0.001). Findings in preeclamptic placentas were identical to those in healthy placentas. After the perfusion, the recovery rates (ie, the sum of the concentrations in the maternal and fetal circulations at 180 minutes versus that at t=0) of the statins and the control substance antipyrine were over 75% under all conditions (Figure S4D and S4E). This indicates that the majority of the statins remained in the perfusion fluid compartment and only a limited amount accumulated in tissue.

In healthy placentas, pravastatin, but not fluvastatin, reduced the release of both chemerin and sFlt-1, while in preeclamptic placentas, both statins reduced the release of chemerin and sFlt-1 (Figure 2). Only pravastatin increased the release of PlGF in preeclamptic placentas, and a similar tendency was observed in healthy placentas. No drug affected the release of chemerin, sFlt-1, and PlGF in the fetal circulation (Figure S3).

### The Effects of Pravastatin and Fluvastatin on Chemerin, sFlt-1, and PlGF in Placental Villous Explants

**Figure 4.**
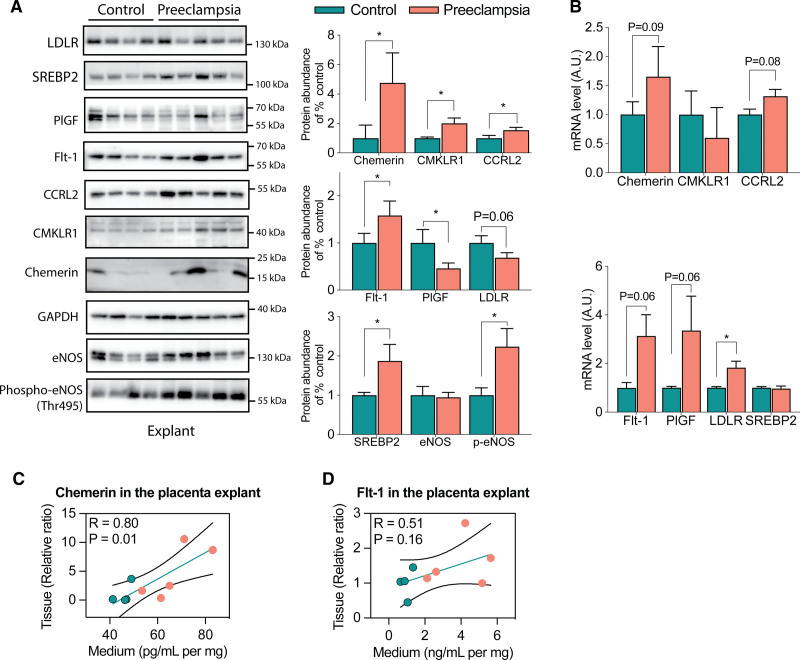
**Protein and mRNA expression in villous explants of healthy and preeclamptic placentas. A**, Western blot data including the quantification of protein abundance following its normalization vs control, except for p-eNOS (Thr495-phosphorylated endothelial nitric oxide synthase), which was normalized vs eNOS (endothelial nitric oxide synthase). **B**, mRNA levels. **C** and **D**, Association between the tissue and medium levels of chemerin and Flt-1 (fms-like tyrosine kinase-1)/sFlt-1 (soluble fms-like tyrosine kinase-1). Data are mean±SEM of n=4 to 6. *P<0.05 vs control. Arbitrary unit (A.U.) is defined as the ratio of mRNA expression to the average expression of the control. CCRL2 indicates CC motif chemokine receptor-like 2; CMKLR1, chemerin chemokine-like receptor 1; LDLR, low-density lipoprotein receptor; PlGF, placental growth factor; and SREBP2, sterol regulatory element-binding protein 2.

Clinical characteristics of the samples that were included in the placental villous explant experiments are provided in Table S6. After 24 hours of incubation without drug exposure, higher protein levels of CMKLR1, CCRL2, SREBP2, Flt-1 (fms-like tyrosine kinase-1), and chemerin were observed in preeclamptic explants compared with healthy control explants (Figure 4A). While total eNOS (endothelial nitric oxide synthase) was not altered, the ratio of Thr495-phosphorylated eNOS versus total eNOS (reflecting eNOS inactivation) was increased in preeclamptic explants. In contrast, PlGF was lower in preeclamptic explants, while a similar lower tendency (*P*=0.06) was observed for the LDLR. Simultaneously, at the mRNA level, chemerin, CCRL2, Flt-1, PlGF, and LDLR showed a tendency for upregulation in preeclampsia explant tissue compared with healthy controls, whereas CMKLR1 and SREBP2 did not (Figure 4B).

**Figure 5.**
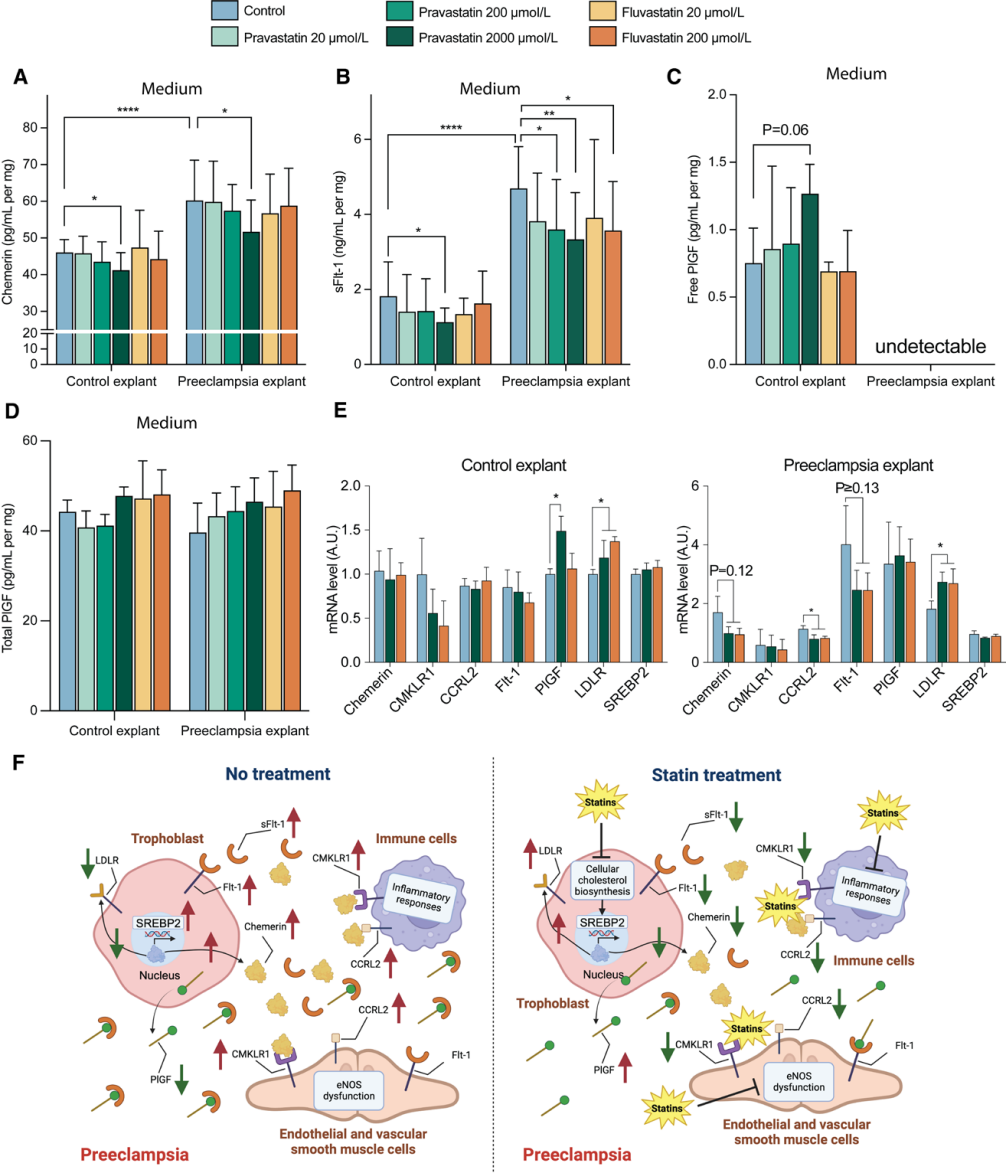
**Effects of pravastatin and fluvastatin on placental explants. A** through **D**, Release of chemerin, sFlt-1 (soluble fms-like tyrosine kinase-1), free PlGF (placental growth factor), and total PlGF into the medium. **E**, mRNA level of chemerin, CMKLR1 (chemerin chemokine-like receptor 1), CCRL2 (CC motif chemokine receptor-like 2), Flt-1, PlGF, low-density lipoprotein receptor (LDLR), and SREBP2 (sterol regulatory element-binding protein 2). Data are mean±SEM of n=4-6. *P<0.05, **P<0.01, and ****P<0.0001 vs control. Arbitrary unit (A.U.) is defined as the ratio of mRNA expression to the average expression of the control. **F**, The potential consequences of statin treatment in preeclampsia.

Preeclamptic explants released more chemerin and sFlt-1 into the medium (Figure 5A and 5B), while free PlGF was detectable only in the medium of healthy explants (Figure 5C). Yet, the levels of total PlGF (ie, free plus sFlt-1-bound PlGF) were identical in the medium of healthy and preeclamptic explants. This indicates that the majority of PlGF in the medium of preeclamptic placentas was sFlt-1-bound. Tissue and medium levels correlated positively in the case of both chemerin (Figure 4C) and Flt-1/sFlt-1 (Figure 4D).

Pravastatin, at a concentration of 2000 μmol/L, reduced the release of chemerin and sFlt-1 and increased the release of free PlGF from control explants into the medium (Figure 5A through 5C), without altering total PlGF in the medium (Figure 5D). At the tissue level, it did not affect the mRNA expression of chemerin, CMKLR1, CCRL2, Flt-1, and SREBP2, but it did upregulate PlGF and the LDLR (Figure 5E). The latter findings were confirmed at the protein level for CCRL2, SREBP2, Flt-1, PlGF, and LDLR, while the protein levels of chemerin and CMKLR1 decreased (Figure S5A). Pravastatin at 2000 μmol/L also downregulated the ratio of Thr495-phosphorylated eNOS versus total eNOS without changing the total eNOS level (Figure S5A). In preeclamptic explants, pravastatin at 2000 μmol/L again reduced the release of chemerin and sFlt-1 into the medium, while free PlGF remained undetectable, and medium total PlGF was unaltered (Figure 5A through 5D). At the tissue level, pravastatin upregulated the LDLR both at mRNA and protein levels, while the opposite was true for CCRL2 (Figure 5E; Figure S5B). Pravastatin additionally downregulated the tissue protein levels of chemerin, CMKLR1, and Flt-1, as well as the Thr495-phosphorylated eNOS/total eNOS ratio, and upregulated the tissue protein level of PlGF (Figure S5B).

Fluvastatin, at its highest tested concentration (200 μmol/L), reduced the release of sFlt-1 into the medium, without affecting chemerin, free PlGF, or total PlGF in both healthy and preeclamptic explants (Figure 5A through 5D). It upregulated the tissue mRNA levels of the LDLR in both types of explants while downregulating the Thr495-phosphorylated eNOS/total eNOS ratio and the tissue protein levels of chemerin and CMKLR1 (Figure 5E; Figure S5). Yet, only in preeclamptic explants, fluvastatin downregulated the CCRL2 mRNA levels (Figure 5E) and the tissue protein levels of Flt-1 (Figure S5B).

Under no condition did pravastatin or fluvastatin alter the medium levels of hCG or LDH, confirming that these drugs did not affect tissue viability (data not shown).

### Wire Myography Experiments

**Figure 6.**
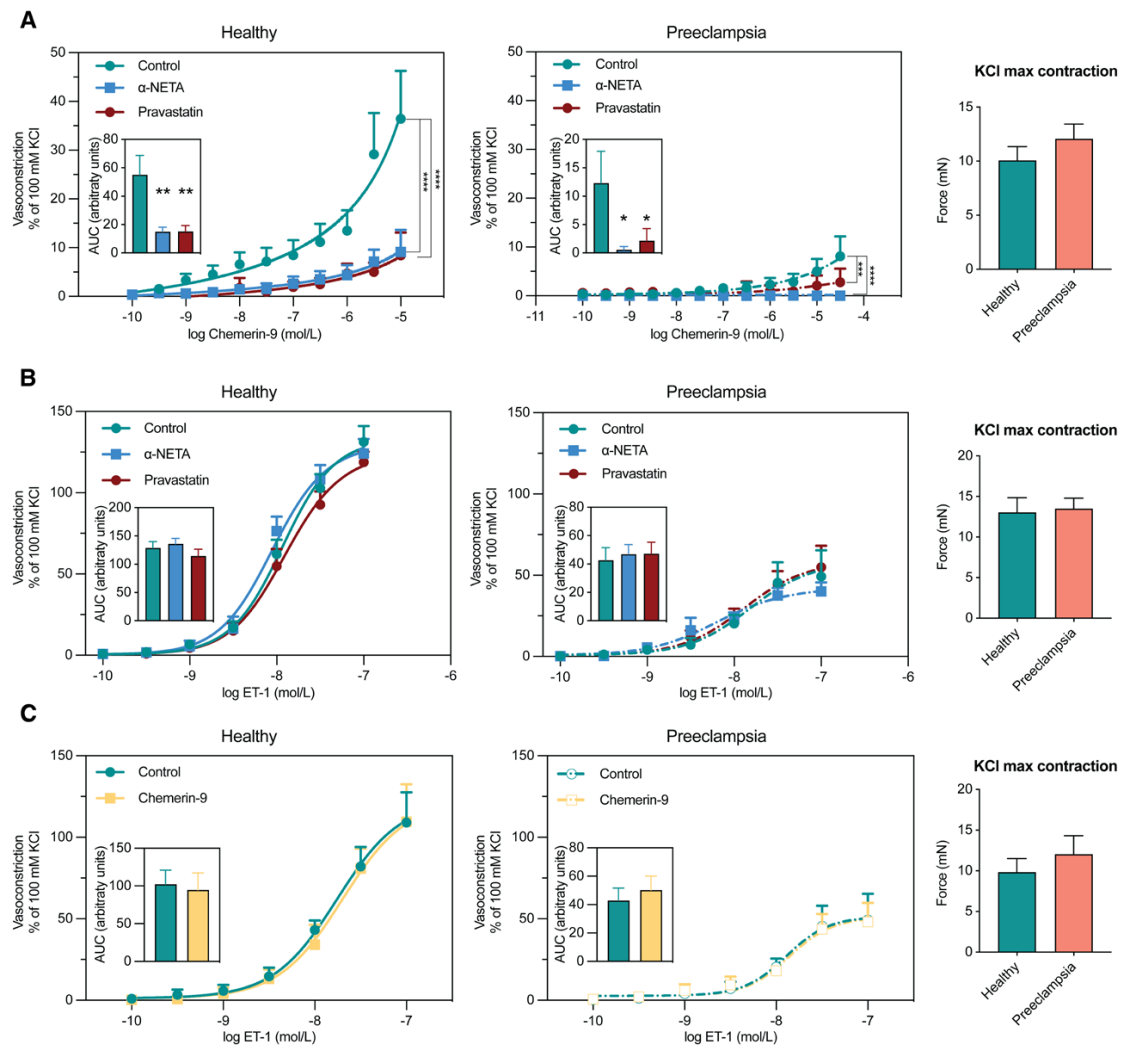
**Response to chemerin-9 and ET-1 (endothelin-1) in chorionic plate arteries from healthy (n=3−10) and preeclamptic (n=3) placentas. A** through **C**, Concentration-response curves were constructed in the absence or presence of the chemerin chemokine-like receptor 1 antagonist α-NETA (2-[a-Naphthoyl]ethyltrimethylammonium iodide), pravastatin, or chemerin-9. Data (mean±SEM) have been expressed as a percentage of the contraction to 100 mmol/L KCl, shown on the right. *P<0.05, **P<0.01, ***P<0.001, and ****P<0.0001 vs control. AUC indicates area under the curve.

Clinical characteristics of the 10 healthy and 3 preeclampsia placentas that were included in the wire myography studies can be found in Table S6. KCl constrictions were identical in both groups (Figure 6). Chemerin-9 constricted both healthy and preeclamptic chorionic plate arteries (Figure 6A) although its effects were much smaller (*P*<0.0001) in the latter. In both groups, pravastatin (20 μmol/L) and the chemerin1/CMKLR1 receptor antagonist α-NETA (3 μmol/L) blocked these constrictor effects. The ET-1-induced constrictor effects were also smaller (*P*<0.01) in preeclamptic chorionic plate arteries (Figure 6B and 6C). The ET-1 effects were not affected by either pravastatin or α-NETA, nor did chemerin-9 preincubation affect the ET-1-induced responses.

## DISCUSSION

This study shows that chemerin levels are elevated in women with preeclampsia, particularly those displaying fetal complications. The addition of chemerin to a traditional prediction model (based on gestational age, parity, proteinuria, and mean arterial blood pressure) improved the prediction of preeclampsia and fetal complications to a similar degree as the sFlt-1/PlGF ratio, and when combined with this ratio, the best prediction rates were achieved. These data suggest that the predictor effects of chemerin are additive to those of the sFlt-1/PlGF pathway. This is further supported by our observation that the correlations of chemerin with creatinine, uric acid, mean arterial pressure, birthweight, and estimated glomerular filtration rate were unaffected after correction for the sFlt-1/PlGF ratio. Our data in perfused placentas reveal that preeclamptic placentas release more chemerin and sFlt-1 and less PlGF compared with healthy placentas. Chemerin in its active form turns out to be a constrictor, and although it has been suggested to additionally facilitate the constrictor effects of other constrictors (including ET-1^16^), we were unable to confirm this concept. Indeed, preincubation of chorionic plate arteries with chemerin-9 did not improve the response to ET-1. Interestingly, pravastatin blocked the constrictor effects of chemerin to the same degree as the chemerin1/CMKLR1 receptor antagonist α-NETA. Moreover, pravastatin, and, to a lesser degree, fluvastatin, suppressed the release of chemerin and sFlt-1 from perfused healthy and preeclamptic placentas, and in preeclamptic placentas, this resulted in a parallel upregulation of the levels of free PlGF. The difference between pravastatin and fluvastatin may relate to the fact that the placental transfer of the latter was around half that of the former. Finally, studies in explants from healthy and preeclamptic placentas confirmed that statins suppress both chemerin and sFlt-1 expression. Collectively, these findings suggest that chemerin could be involved in the pathology of preeclampsia, while pravastatin and fluvastatin might serve as a potential treatment for preeclampsia, among others by suppressing chemerin (Figure 5F).

Elevated chemerin levels have been observed previously in preeclampsia cohorts of small size,^14,41^ and animal data have demonstrated that placental chemerin overexpression during pregnancy results in hypertension, fetal growth restriction, and maternal renal dysfunction.^10^ The present clinical data fully confirm these findings. They also confirm the positive association between serum chemerin and the sFlt-1/PlGF ratio,^10^ a biomarker currently used to predict preeclampsia.^42^ However, despite this correlation, chemerin per se displayed additive predictor effects on top of the sFlt-1/PlGF ratio, suggesting that its effects are not necessarily fully identical to those of the sFlt-1/PlGF pathway.

Circulating chemerin is, at least partly, adipose tissue-derived, and this may explain its correlation with BMI.^6,7^ However, the placenta is another well-known source of chemerin, and thus, its upregulated levels in preeclampsia are likely of placental origin.^10,13^ Indeed, the elevated chemerin release from preeclamptic placentas supports this concept. Moreover, in agreement with a previous study,^10^ we observed that the chemerin upregulation in preeclampsia was BMI-independent. Because sFlt-1 is also placenta-derived,^43^ a correlation between chemerin and sFlt-1 is not surprising. In contrast with sFlt-1, PlGF explant content and release from preeclamptic placentas were low, and when measuring free PlGF in the medium of placenta explants from preeclamptic placentas, we were unable to detect it, despite the fact that PlGF mRNA expression was higher in such explants compared with healthy explants. Heating samples at 70 °C is an accepted method to destroy sFlt-1,^36^ thereby liberating sFlt-1-bound PlGF. When applying this to our samples, PlGF became detectable in the medium of preeclamptic explants, at levels that were identical to those in healthy explants. This indicates that the majority of released PlGF in preeclamptic conditions is sFlt-1-bound.

Our data with the chemerin1/CMKLR1 receptor antagonist α-NETA are the first to demonstrate that chemerin contracts placental chorionic plate arteries via CMKLR1 in both healthy and preeclamptic conditions. Although pravastatin was also capable of blocking this constriction, it seems unlikely that it acted as a CMKLR1 antagonist. Pravastatin has been reported to improve placental blood flow in preeclamptic patients.^44^ The placental villous explant data in the current study now show that pravastatin increases eNOS activity. This might counteract the previously reported decrease in eNOS activity and NO production following CMKLR1 activation by chemerin^45^ and could, thus, explain the blocking effects of pravastatin versus chemerin. We were unable to observe the effects of either chemerin, pravastatin, or α-NETA on ET-1-induced vasoconstriction. Possibly, the ET-1-enhancing effects of chemerin that have been reported before require a longer preincubation.^46^ Interestingly, all contractile responses in preeclamptic vessels were reduced. To what degree this relates to the proinflammatory condition in this disorder needs to be investigated further.

Pravastatin and, to a lesser degree, fluvastatin, when used at clinically relevant concentrations,^28,29^ acutely reduced the placental release of both sFlt-1 and chemerin, both in healthy and preeclamptic placentas. Consequently, the free levels of PlGF increased. A large clinical trial where pravastatin 20 mg/day was applied to women at high risk of preeclampsia from 35 to 37 weeks of gestation until delivery did not reveal such effects in vivo,^3^ while in our placental villous explant model, we only observed sFlt-1 and chemerin reductions at a concentration of 2000 μmol/L. Thus, either pravastatin needs to be dosed higher (40 mg/day), or its in vivo exposure needs to be started earlier. Here, we note that although the current recommendation according to the major international guidelines is to stop statins during pregnancy, the US Food and Drug Administration has removed the strongest warning and provides some opening to continue statin therapy during pregnancy if the benefit outweighs the risk.^47^ In addition, given that pravastatin is a hydrophilic statin, its vascular permeability might be less than that of lipophilic statins such as fluvastatin.^48,49^ Yet, if anything, we found that pravastatin transferred at 2-fold higher levels to the fetal compartment than fluvastatin, and thus, its placental transfer not only relies on lipophilicity. Moreover, the effects of pravastatin generally were stronger than those of fluvastatin, which, at least in part, might relate to its greater transfer.

To investigate why statins lower chemerin, we focused on the LDLR. Both LDLR and chemerin expression rely on the transcription factor SREBP2.^9,50^ We observed that preeclamptic explants contained more SREBP2 and less LDLR at the protein level. Theoretically, this might shift the balance in favor of chemerin. Subsequently, treatment with a statin upregulated the LDLR mRNA levels, thereby diminishing SREBP2 availability for chemerin transcription. Yet, other factors that upregulate SREBP2 include tumor necrosis factor-α, interleukin-1β, and nuclear factor kappa B. These factors are upregulated in preeclampsia and can be inhibited by statins, and thus, their suppression might also explain why statins would lower chemerin.^51–55^

A statin-induced reduction of chemerin would affect the downstream effects of chemerin on inflammation, endothelial dysfunction, and spiral remodeling. Combined with an improved sFlt-1/PlGF ratio, this would offer an attractive mechanism by which statins may prevent or treat preeclampsia at an early stage, also considering that these drugs are not considered teratogenic.^56,57^

## PERSPECTIVES

In this study, we show that chemerin is increased in both serum and placentas of women with preeclampsia. As such, it might serve as a biomarker for preeclampsia and its fetal complications on top of the sFlt-1/PlGF ratio. Chemerin not only acts as a proinflammatory adipokine^6,7^ but also exerts constrictor effects. Interestingly, statins suppressed the placental release of chemerin, as well as its constrictor effects, and these effects were the largest for a statin that passed the placental barrier best. Statins also suppressed the release of sFlt-1 and, thus, upregulated free PlGF. The mechanism underlying these phenomena may relate to the capacity of statins to upregulate NO production and the LDLR. In summary, this study offers preliminary ex vivo and in vitro data about the potential of statins to treat preeclampsia and its deleterious consequences by lowering chemerin.

## ARTICLE INFORMATION

### Acknowledgments

The authors thank people who participated in this study and kindly donated tissues.

### Sources of Funding

L. Tan and K. Verdonk were supported by the Stichting Lijf en Leven. E.O. Cruz-López was supported by the Consejo Nacional de Ciencia y Tecnología, Mexico (grant 739513).

### Disclosures

None.

### Supplemental Material

Tables S1–S6

Figures S1–S5

## Supplementary Material
