# Supplementary material for: Statins Prevent the Deleterious Consequences of Placental Chemerin Upregulation in Preeclampsia
Source: Hypertension. 2024 Feb 16;81(4):861–75. doi: 10.1161/HYPERTENSIONAHA.123.22457 (PMC10956680; doi:10.1161/HYPERTENSIONAHA.123.22457)
Supplement: Supplementary file 1 [file hyp-81-861-s001.doc]

**Statins prevent the deleterious consequences of placental chemerin upregulation in preeclampsia**

Lunbo Tan^1,2^, Ans C.M. Kluivers^1,3^, Edwyn O. Cruz-López^1^, Michelle Broekhuizen^1,4^, Zhongli Chen^5^, Rugina I. Neuman^1,3^, Sam Schoenmakers^3^, Liesbeth Ruijgrok^6^, Daan van de Velde^6^, Brenda C.M. de Winter^6^, Antoon J. van den Bogaerdt^7^, Xifeng Lu^2^, A.H. Jan Danser^1^, Koen Verdonk^1*^

^1^Division of Vascular Medicine and Pharmacology, Department of Internal Medicine, Erasmus MC, Rotterdam, The Netherlands.

^2^Clinical Research Center, The First Affiliated Hospital of Shantou University Medical College, Shantou 515041, China.

^3^Department of Obstetrics and Gynaecology, Erasmus MC, Rotterdam, The Netherlands.

^4^Division of Neonatology, Department of Neonatal and Pediatric Intensive Care, Erasmus MC, Rotterdam, the Netherlands

^5^Department of Internal Medicine, Academic Center for Thyroid Diseases, Erasmus MC, Rotterdam, The Netherlands.

^6^Department of Hospital Pharmacy, Erasmus MC, Rotterdam, The Netherlands.

^7^ETB-BISLIFE, Heart Valve Department, Beverwijk, The Netherlands.

^*^Correspondence to: Koen Verdonk, MD, PhD. Email: k.verdonk@erasmusmc.nl.

**Supplemental Information**

**Table S1. List of the primary antibodies**

| Antibody | Dilution | Vender | Catalog no. |
| --- | --- | --- | --- |
| Anti-Chemerin antibody | 1:500 | Thermo Fisher | MA5-43890 |
| Anti-Chemerin antibody | 1:1000 | R&D SYSTEMS | MAB2324 |
| Anti-CMKLR1 antibody | 1:500 | Abcam | ab230442 |
| Anti-CCRL2 antibody | 1:1000 | Proteintech | 66611-1-Ig |
| Anti-Flt-1 antibody | 1:1000 | Proteintech | 13687-1-AP |
| Anti-PlGF antibody | 1:1000 | Abcam | ab196666 |
| Anti-LDLR antibody | 1:1000 | Proteintech | 10785-1-AP |
| Anti-SREBP2 antibody | 1:1000 | Proteintech | 28212-1-AP |
| Anti-GAPDH antibody | 1:5000 | Proteintech | 60004-1-Ig |
| Anti-eNOS antibody | 1:1000 | Proteintech | 27120-1-AP |
| Anti-Phospho-eNOS antibody | 1:1000 | Proteintech | 28939-1-AP |

**Table S2. Human primer sequences.**

| Gene | Primer sequences (5’-3’) |
| --- | --- |
| RARRES2  (retinoic acid receptor responder protein 2, chemerin) | Forward: TGGAAGAAACCCGAGTGCAAA |
|  | Reverse: AGAACTTGGGTCTCTATGGGG |
| LDLR (low density lipoprotein receptor) | Forward: CTACAAGTGGGTCTGCGATG |
|  | Reverse: TTTGCAGGTGACAGACAAGC |
| 36B4 (acidic ribosomal phosphoprotein P0) | Forward: TCTACAACCCTGAAGTGCTTGAT |
|  | Reverse: CAATCTGCAGACAGACACTGG |
| YWHAZ (tyrosine 3-monooxygenase) | Forward: ACTTTTGGTACATTGTGGCTTCAA |
|  | Reverse: CCGCCAGGACAAACCAGTAT |
| CMKLR1 (chemerin chemokine-like receptor 1) | Forward: GAGGCGTGACATAGAATGGA |
|  | Reverse: TGATATGGATTGGGAGGAAGAC |
| CCRL2 (CC motif chemokine receptor like 2) | Forward: GAGGCAGAGCAATGTGACAA |
|  | Reverse: ATTTTCCACGCGTTTGAGTC |
| PlGF (placental growth factor) | Forward: ATGTTCAGCCCATCCTGTGT |
|  | Reverse: CTTCATCTTCTCCCGCAGAG |
| Flt-1 (fms-like tyrosine kinase-1) | Forward: CAGCGCATGGCAATAATAGA |
|  | R: GCTATGCAAATGTAGATTCCAGAAA |
| SREBP2 (sterol regulatory element-binding protein 2) | Forward: AACGGTCATTCACCCAGGTC |
|  | Reverse: GGCTGAAGAATAGGAGTTGCC |
| β-actin | Forward: CTCCCTGGAGAAGAGCTACG |
|  | Reverse: GAAGGAAGGCTGGAAGAGTG |

**Table S3. Characteristics of study participants at time of blood sampling, according to whether they had preeclampsia.**

| Factors | No preeclampsia | Preeclampsia | P-value |
| --- | --- | --- | --- |
|
| n | 366 | 101 |  |
| Maternal age (years) | 31 (27-35) | 31 (27-35) | NS |
| Caucasian | 264 (72%) | 68 (67%) | NS |
| African | 61 (17%) | 25 (25%) | NS |
| Other ethnicity | 41 (11%) | 8 (8%) | NS |
| Gestational age at sampling (weeks) | 36.1 (32.1-38.0) | 30.6 (27.6-34.1) | P<0.001 |
| Pre-pregnancy BMI (kg/m²) | 26.0 (23.0-30.73) | 27.1 (21.7-30.0) | NS |
| Nulliparous | 206 (56.3%) | 63 (62.4%) | NS |
| History of preeclampsia | 49 (13%) | 15 (15%) | NS |
| History of smoking | 22 (6.25%) | 6 (6.59%) | NS |
| Gestational age at delivery (weeks) | 38.5 (37.2-39.6) | 32.0 (29.3-36.1) | P<0.001 |
| Birthweight (g) | 3235 (2800-3614) | 1435 (1050-2480） | P<0.001 |
| sFlt-1/PlGF ratio | 23.0 (6.0-52.0) | 90.0 (27.0-317.5) | P<0.001 |
| Creatinine (mmol/L) | 56 (50-62) | 60 (52-70) | P=0.001 |
| Uric acid (mmol/L) | 0.27 (0.23-0.32) | 0.37 (0.26-0.46) | P<0.001 |
| Alanine aminotransferase (U/L) | 14 (10-19) | 26 (17-89) | P<0.001 |
| Proteinuria (mg/24 hours) | 180 (110-363) | 900 (525-2699) | P<0.001 |
| Mean arterial pressure (mmHg) | 100 (94-108) | 113 (107-120) | P<0.001 |
| eGFR (mL/min/1.73m^2^) | 118 (109-126) | 101 (82-127) | P=0.005 |
| Chemerin (ng/mL) | 185.4 (142.0-239.2) | 231.6 (167.7-316.7) | P<0.001 |

Data are presented as median (interquartile range) or number (%). BMI = body mass index; eGFR = estimated glomerular filtration rate; sFlt-1/PlGF = soluble Fms‐like tyrosine kinase‐1 / placental growth factor.

**Table S4.** Correlation between clinical parameters and serum chemerin (n=467).

| Factors | Crude model | | Adjusted Model | |
| --- | --- | --- | --- | --- |
|  | R | P-value | R | P-value |
| Maternal age (years) | -0.01 | NS | 0.05 | NS |
| Gestational age at delivery (weeks) | -0.06 | NS | -0.07 | NS |
| Birthweight (g) | -0.11 | P=0.027 | -0.11 | P=0.029 |
| Creatinine (mmol/L) | 0.16 | P=0.002 | 0.20 | P<0.001 |
| Uric acid (mmol/L) | 0.26 | P<0.001 | 0.21 | P<0.001 |
| Alanine aminotransferase (U/L) | 0.04 | NS | -0.04 | NS |
| Proteinuria (mg/24 hours) | 0.20 | P=0.039 | 0.06 | NS |
| Mean arterial pressure (mmHg) | 0.14 | P=0.006 | 0.13 | P=0.034 |
| eGFR (mL/min/1.73m^2^) | -0.27 | P<0.001 | -0.30 | P<0.001 |
| Gestational age at sampling (weeks) | 0.02 | NS | 0.04 | NS |
| Pre-pregnancy BMI (kg/m²) | 0.03 | NS | -0.01 | NS |
| sFlt-1/PlGF ratio | 0.20 | P<0.001 |  |  |

eGFR = estimated glomerular filtration rate; sFlt-1/PlGF = soluble Fms‐like tyrosine kinase‐1 / placental growth factor; Crude model: correlation with chemerin; Adjusted Model: correlation with chemerin after correction for the sFlt-1/PlGF ratio.

**Table S5.** Characteristics of the patients whose placentas were perfused with either no drug, pravastatin, or fluvastatin.

| Factors | Healthy | | | Preeclampsia | | |
| --- | --- | --- | --- | --- | --- | --- |
|  | No drug | Pravastatin | Fluvastatin | No drug | Pravastatin | Fluvastatin |
|  |  |  |  |  |  |  |
| N | 10 | 5 | 5 | 5 | 3 | 3 |
| Maternal age | 33 (31-37) | 34 (32-38) | 34 (26-38) | 31 (28-35) | 29 (28-30) | 29 (28-30) |
| Caucasian | 8 | 2 | 2 | 2 | 2 | 1 |
| African | 1 | 2 | 2 | 1 | 1 | 0 |
| Other ethnicity | 1 | 1 | 1 | 2 | 0 | 2 |
| Fetal sex (n female/male) | 13/9 | 3/2 | 4/1 | 2/3 | 1/2 | 1/2 |
| Gestational age at delivery (weeks) | 39.0  (38.4-39.2) | 39.1  (38.8-39.2) | 38.6  (38.5-39.2) | 32.0 *  (31.2-33.5) | 30.4 *  (29.4-30.4) | 30.0 *  (26.6-31.1) |
| Pre-pregnancy BMI (kg/m^2^) | 24.3  (22.5-26.0) | 23.9  (21.4-26.1) | 23.5  (21.0-37.8) | 27.6  (23.5-35.1) | 30.9  (22.0-33.2) | 28.6  (21.5-29.9) |
| Parity, n | 1 (0-2) | 2 (1-2) | 1 (0-2) | 0 (0-3) | 0 (0-2) | 0 (0-2) |
| Mean arterial pressure (mmHg) | 91.5  (88.0-97.5) | 93.0  (80.5-98.0) | 88.0  (85.0-93.0) | 122.0 *  (119.0-134.5) | 125.0 *  (122.0-133.0) | 133.0 *  (117.0-137.0) |
| Birth weight (g) | 3738  (3178-3800) | 3703  (3421-3969) | 3415  (3134-3501) | 1305 *  (1131-1809) | 1040 *  (1015-1775) | 1170 *  (750-1320) |
| Placenta weight (g) | 618  (544-719) | 632  (507-670) | 783  (645-794) | 344 *  (286-407) | 310 *  (258-379) | 342 *  (165-360) |

Data are presented as median (interquartile range) or number. BMI = body mass index. *P<0·05 versus the no drug group of the healthy placentas.

**Table S6.** Clinical data of the placental explant and wire myography experiments.

| Factors | Explants | | Wire myography | |
| --- | --- | --- | --- | --- |
|  | Healthy | Preeclampsia | Healthy | Preeclampsia |
|  |  |  |  |  |
| N | 4 | 6 | 10 | 3 |
| Maternal age | 33 (32-34) | 29 (28-39) | 33 (29-38) | 36 (31-40) |
| Caucasian | 2 | 3 | 6 | 2 |
| African | 2 | 1 | 1 | 0 |
| Other ethnicity | 0 | 2 | 3 | 1 |
| Fetal sex (n female/male) | 1/3 | 2/4 | 6/4 | 1/2 |
| Gestational age at delivery (weeks) | 39.0 (37.8-39.2) | 27.3* (25.9-29.4) | 39.0 (38.4-39.1) | 33.2** (30.6-35.2) |
| Pre-pregnancy BMI (kg/m^2^) | 27.3 (22.6-30.0) | 27.4 (22.0-30.3) | 23.5 (21.5-26.3) | 28.6 (21.3-30.4) |
| Parity, n | 1 (0-1) | 0 (0-2) | 1 (1-2) | 1 (0-2) |
| MAP (mmHg) | 83.5 (82.3-91.5) | 135.5* (126.5-154.5) | 89.0 (85.8-99.3) | 133.0** (113.0-138.0) |
| Birth weight (g) | 3728 (2994-3925) | 725* (603-916) | 3445 (3135-3715) | 1270** (1170-2480) |
| Placenta weight (g) | 650 (507-684) | 247* (219-315) | 518 (423-759) | 288* (231-521) |

Data are presented as median (interquartile range) or number. BMI = body mass index. MAP = mean arterial pressure. *p<0·05, ** P<0.01 versus the healthy placentas.

**
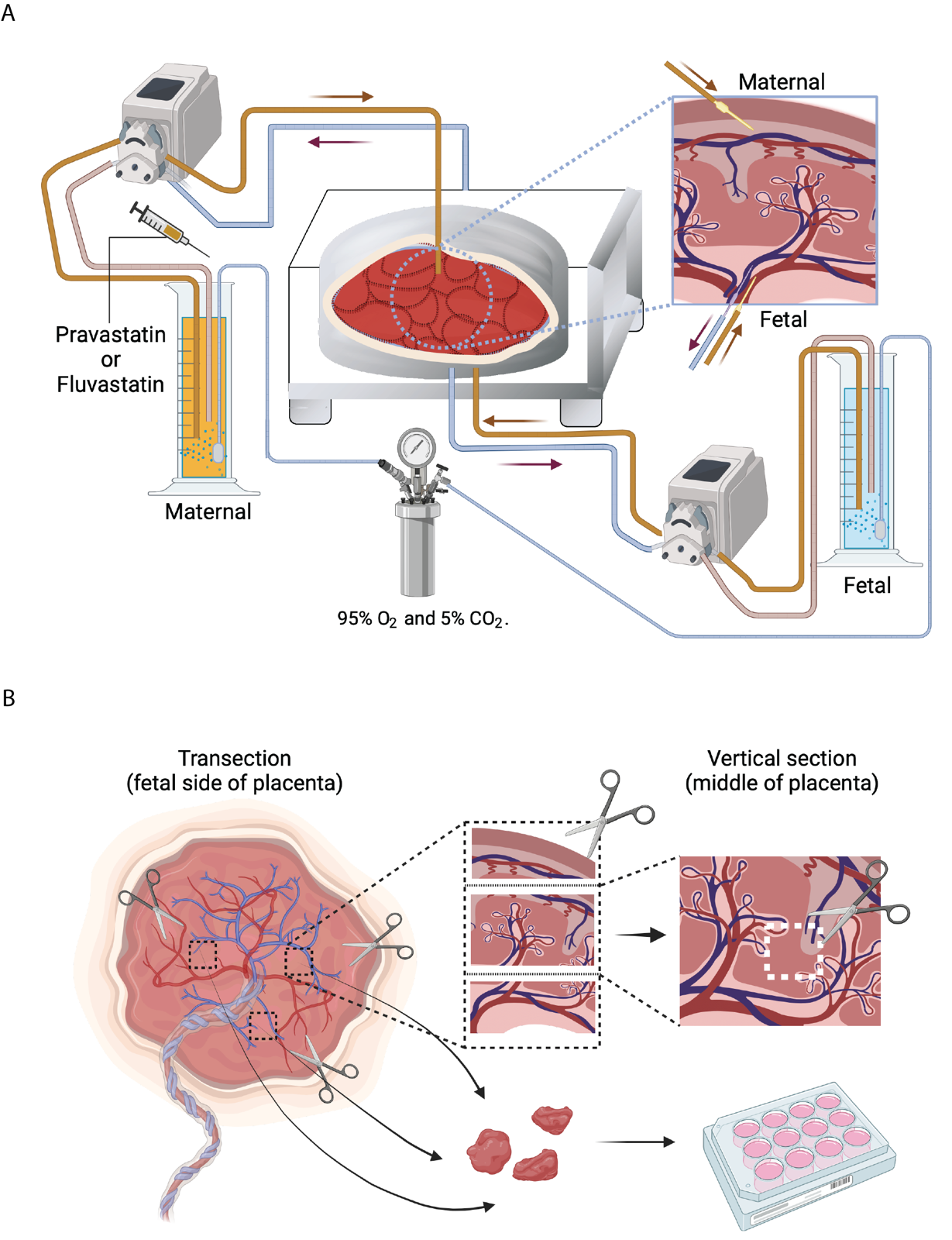
**

**Figure S1. Schematic diagram of the ex vivo dual-sided placental cotyledon perfusion approach (A) and the villous explant culture (B).**

**
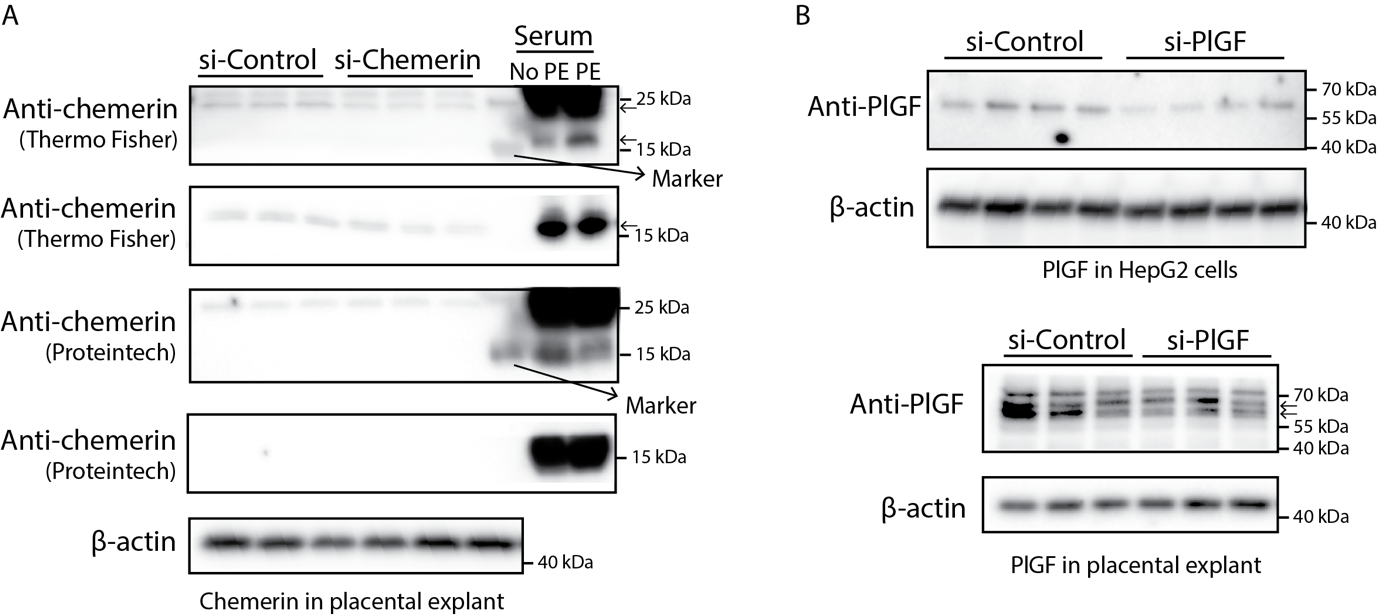
**

**Figure S2. Western blot results for chemerin (A) and placental growth factor (PlGF, B), obtained in placental explants and HepG2 cells, using serum from both preeclamptic (PE) and non-preeclamptic (no PE) patients as positive control.** For chemerin 2 different primary antibodies were used, one from Thermo Fisher and one from Proteintech. Only with the Thermo Fisher antibody did the chemerin signal go down after knockdown with small interfering (si) RNA. See text for further explanation.


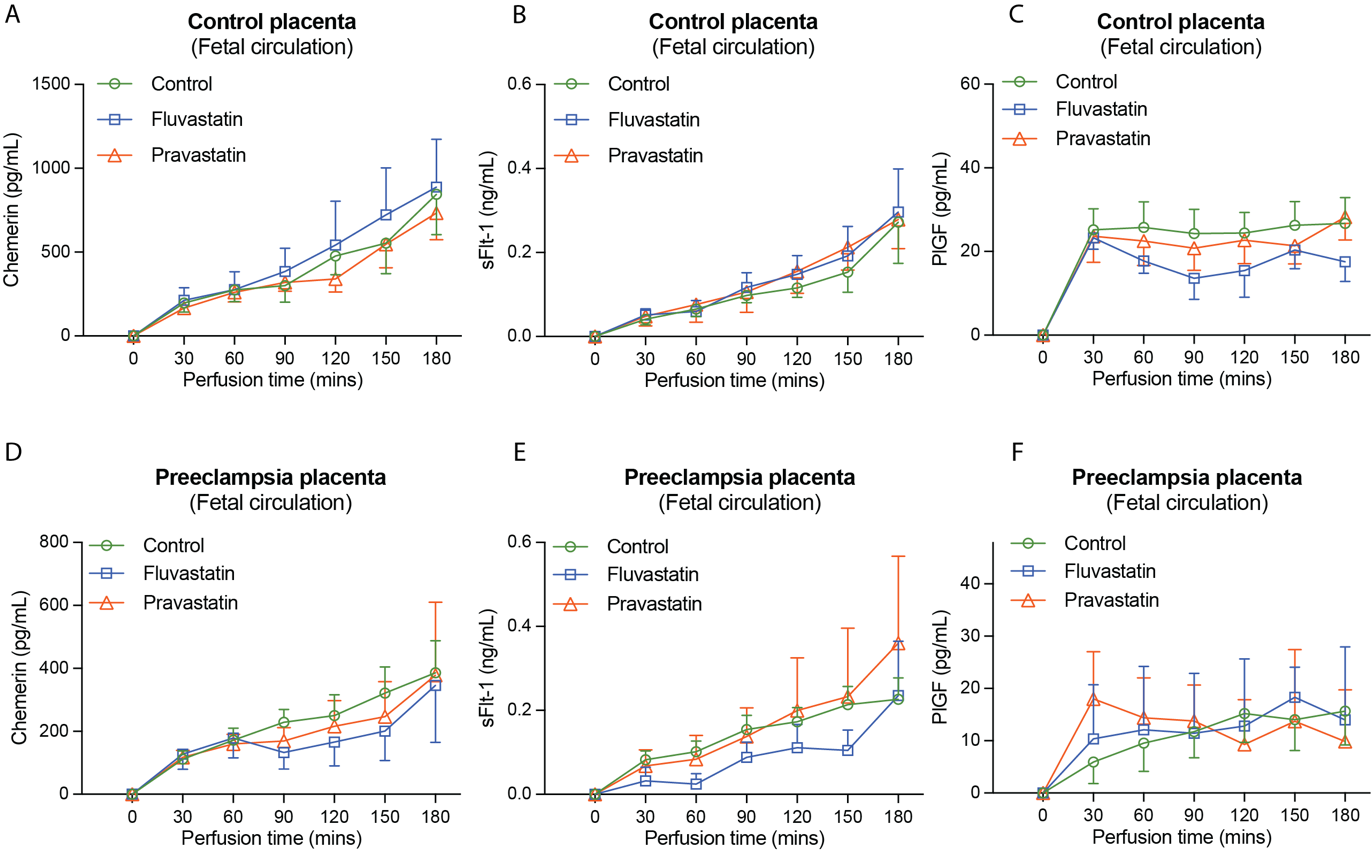


**Figure S3. Fetal release of chemerin, sFlt-1 (soluble Fms-like tyrosine kinase-1), and PlGF (placental growth factor) from healthy and preeclamptic placentas during vehicle or statin perfusion.** (**A-C**) Concentrations of chemerin, sFlt-1 and PlGF in the fetal effluent of healthy placentas perfused with vehicle (control; n=10), 5 mg/L fluvastatin (n=4) or 1 mg/L pravastatin (n=5). **(D-F)** Concentrations of chemerin, sFlt-1 and PlGF in the fetal effluent of preeclamptic placentas perfused with vehicle (control; n=5), 5 mg/L fluvastatin (n=3) or 1 mg/L pravastatin (n=3). Data are mean ± SEM, sFlt-1/PlGF, soluble Fms‐like tyrosine kinase‐1/placental growth factor.


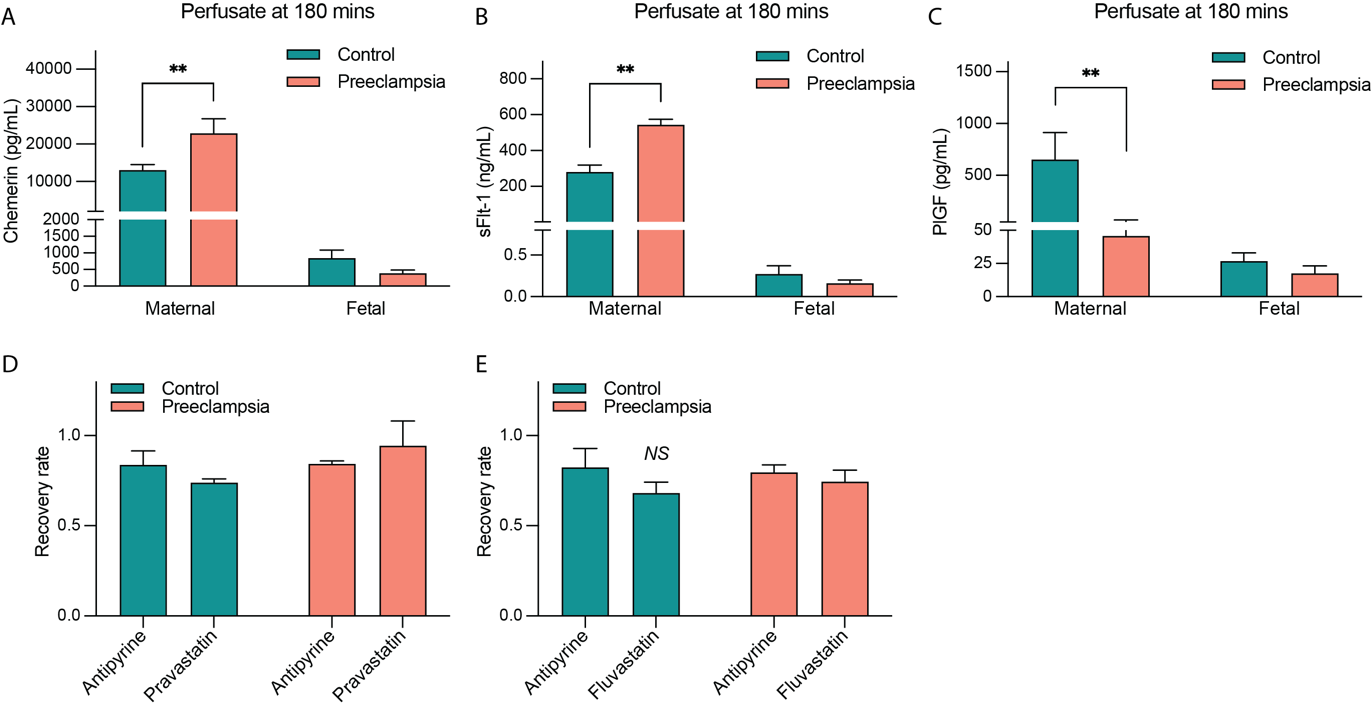


**Figure S4. Placental perfusion with pravastatin or fluvastatin. (A-C)** The maternal perfusate of preeclamptic placentas (n=5) contains more chemerin and soluble Fms-like tyrosine kinase-(sFlt-1) and less placental growth factor (PlGF) than the maternal perfusate of healthy (control) placentas (n=10). This is not the case for the fetal perfusate. **(D, E)** Recovery rate of antipyrine and the applied statin after perfusion with either pravastatin or fluvastatin in healthy and preeclampstic placentas, calculated as the sum of the maternal and fetal levels at t=180 minutes versus the sum of the maternal and fetal levels at t=0. Data are mean ± SEM of n=3-6, *P<0.05, **P<0.01 versus control.


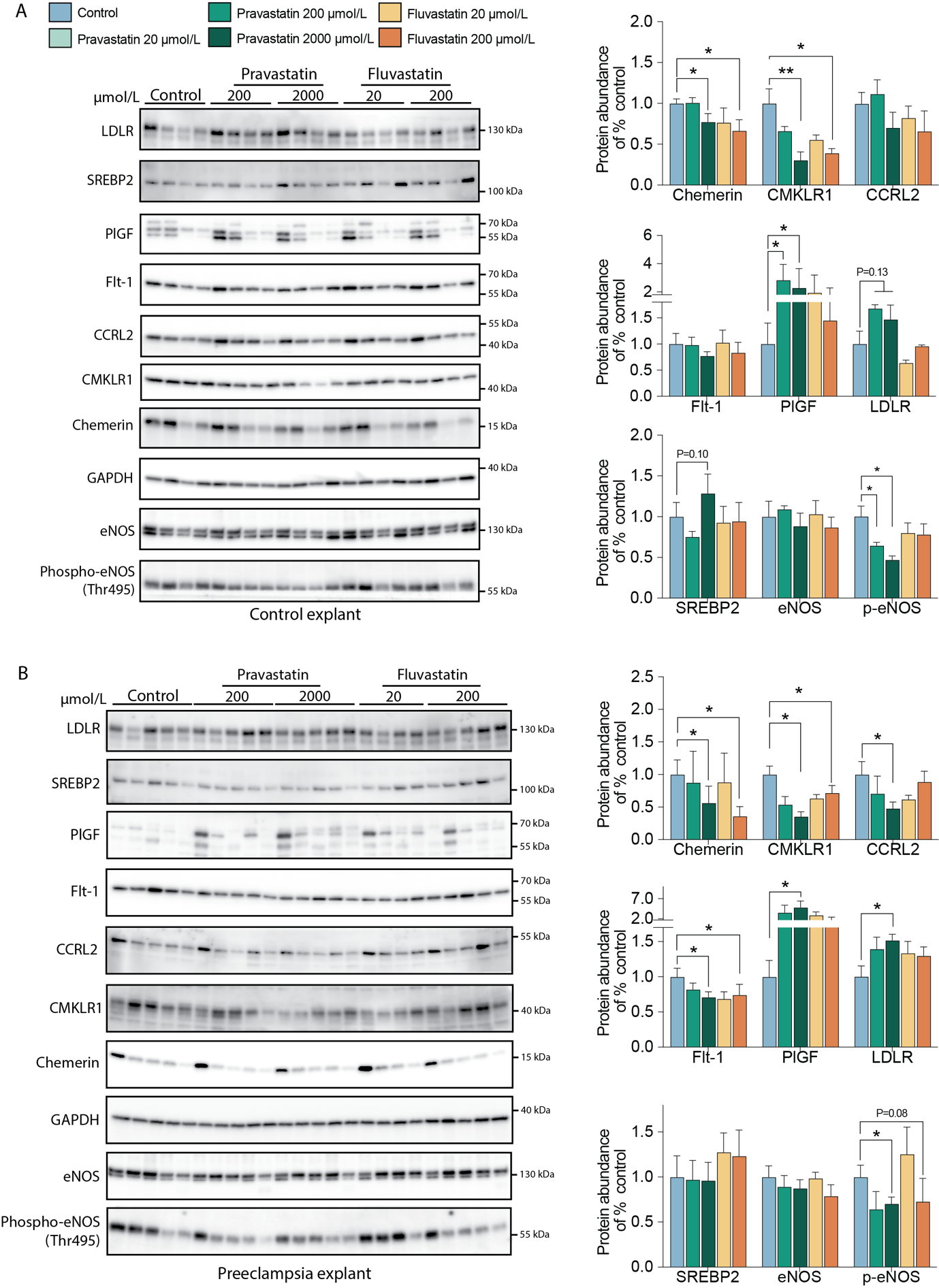


**Figure S5. Effects of pravastatin and fluvastatin on placental explants.** Western blot data including the quantification of protein abundance following its normalization versus control in healthy **(A)** and preeclamptic **(B)** explants, except for p-eNOS, which was normalized with eNOS. CMKLR1, chemerin chemokine-like receptor 1 (CMKLR1); CCRL2, CC motif chemokine receptor like 2; Flt-1, Fms-like tyrosine kinase-1, PlGF, placental growth factor (PlGF); LDLR, low-density lipoprotein receptor (LDLR); eNOS, endothelial NO synthase; p-eNOS, Thr495-phosphorylated eNOS; SREBP2, sterol regulatory element-binding protein 2. Data are mean ± SEM of n=4-6. *P<0.05, **P<0.01 versus control.
